# A Randomized Controlled Trial Comparing Efficacy and Safety of Antidepressant Monotherapy

**DOI:** 10.7759/cureus.59074

**Published:** 2024-04-26

**Authors:** N. Simple Santi, Sashi B Biswal, Birendra Narayan Naik, Jyoti Prakash Sahoo, Bhabagrahi Rath

**Affiliations:** 1 Pharmacology, Veer Surendra Sai Institute of Medical Sciences and Research, Burla, IND; 2 Psychiatry, Veer Surendra Sai Institute of Medical Sciences and Research, Burla, IND; 3 Pharmacology, Kalinga Institute of Medical Sciences, Bhubaneswar, IND

**Keywords:** adverse event, montgomery-asberg depression rating scale, hamilton depression rating scale, randomized trial, vortioxetine, escitalopram, vilazodone, antidepressant drug, serotonin dysfunction, depressive disorder

## Abstract

Background and objectives: The majority of mainstream antidepressants lack the promise of complete amelioration of symptoms. Other pitfalls include the latency period and side effects. These issues prompted investigations concerning the various roles of serotonin (5-HT) neurotransmissions in the etiology of depression. In this study, each study participant received vilazodone, vortioxetine, and escitalopram monotherapy for major depressive disorder (MDD) for 16 weeks. After that, the subject's scores on the Hamilton Depression Rating Scale (HDRS)-17 item version and the Montgomery Åsberg Depression Rating Scale (MADRS) were evaluated. In the study population, we kept track of the incidence of adverse events.

Methods: Ninety-six patients with MDD participated in this open-label, randomized, three-arm study. Participants were allotted into three groups according to a 1:1:1 ratio and given vilazodone (20-40 mg/day), vortioxetine (5-20 mg/day), or escitalopram (10-20 mg/day) for 16 weeks. Vortioxetine and vilazodone are test medications, with escitalopram serving as the control. After the baseline visit, follow-up appointments were scheduled every four weeks. Per-protocol (PP) and intent-to-treat (ITT) populations served as means for efficacy and safety evaluations, respectively. We prospectively registered this research in the Clinical Trial Registry, India (CTRI) (2022/07/043808).

Results: Out of the 134 patients we screened, 109 (81.34%) were eligible. Ninety-six (88.07%) of them completed the 16-week trial. In the PP population (n = 96), we analyzed efficacy. They had a mean age of 46.3 ± 6.2 years. At baseline, each group's median HDRS score was 30.0 (p = 0.964). Following 16 weeks of antidepressant therapy, these scores dropped to 15.0, 14.0, and 13.0 (p = 0.002). Baseline MADRS scores for all groups were 36.0 (p = 0.741). They had corresponding values of 20.0, 18.0, and 17.0 at 16 weeks (p < 0.001). Regarding both efficacy endpoints, the post-hoc analysis with the Bonferroni correction demonstrated statistically significant differences (p < 0.001). We performed the safety assessments within our ITT population (n = 109). Ninety-six adverse events were recorded. Nonetheless, none of them seemed serious. Still, five participants opted out because of their side effects. Vomiting and nausea were the most frequent side effects.

Conclusion: Compared to escitalopram and vilazodone, vortioxetine demonstrated a statistically significant reduction in HDRS and MADRS scores. It also had fewer and milder side effects*. *We recommend conducting studies involving a broader population to investigate the antidepressant effects of these medications further.

## Introduction

Depressive disorder constitutes one of the most crippling disorders across the globe, exhibiting counterproductive impacts on general well-being, cognitive function, quality of life, and work efficiency [1]. A reported number of 322 million individuals across borders suffer from depression [2,3]. The age distribution of the growing prevalence is not uniform, with the most significant increase spotted in younger individuals [1]. Most of the suicide victims endure symptoms of major depressive disorder (MDD) [4]. Between 1990 and 2017, the worldwide incidence of depressive disorders expanded by 49.86% [5]. Depressive disorders affect 15.9% of Indians annually, according to recent studies [6,7]. MDD has detrimental effects on metabolic, mental, and social health [7-9].

The best medication for the complete remission of MDD is still unknown, even with the broad palette of antidepressants available. It is pertinent to conventional antidepressant medications' side effects and resistance [10]. As a result, there is debate about the issue of whether recently developed drugs should be prescribed for MDD instead of the existing category of antidepressants. One of the selective serotonin receptor inhibitors (SSRIs) that are commonly prescribed for amelioration of MDD is escitalopram. It has established modulatory actions at the allosteric binding site of the serotonin transporter [11]. The SSRI with additional partial agonistic activity at 5-HT_1A_ receptors is vilazodone [12]. Vortioxetine influences serotonin receptor function and interferes with serotonin transporters. When compared to traditional antidepressants, it has demonstrated promising results as a feasible substitute for addressing depression [13]. The hypothesis supporting the current study was that antidepressants with distinct pharmacological mechanisms could offer individuals with MDD an enticing treatment option. This study’s interim analysis showed these drugs' efficacy and safety [3,7,9,14]. Several trials and meta-analyses favored these drugs [15-18].

The purpose of this study was to evaluate the efficacy as well as safety of antidepressant monotherapy using vilazodone, escitalopram, and vortioxetine for 16 weeks. The objectives included evaluating the Hamilton Depression Rating Scale (HDRS) and Montgomery Åsberg Depression Rating Scale (MADRS) scores and tracking the adverse event pattern [19,20].

## Materials and methods

Study design

This was a three-arm, open-label, randomized, active-controlled trial. We gauged the safety and efficacy of monotherapy of vilazodone, vortioxetine, and escitalopram on individuals diagnosed with MDD. The study was conducted at Veer Surendra Sai Institute of Medical Sciences and Research (VIMSAR), Burla, India, between July 2022 and December 2023. Before commencing the trial, we received ethical permission from our Institutional Ethics Committee (029-2022/I-S-T/03). All participants provided consent through themselves or their close relatives before enrollment. Our study was prospectively enlisted in the Clinical Trial Registry, India (CTRI) (2022/07/043808). The study followed institutional guidelines, good clinical practices, and the Declaration of Helsinki.

Study criteria

Our study included females and males diagnosed with MDD and an HDRS score >24. Any documented allergy to study medications, organic brain diseases, psychotic symptoms, abnormal lipid profiles, chronic renal failure, and any thrombo-ischemic event that occurred within the previous six months were all considered exclusion criteria. Moreover, nursing mothers and pregnant women were excluded from this study. The participants could revoke their consent at any time.

Study procedure

This study featured escitalopram as the control and vortioxetine and vilazodone as the test medications. Randomization was done by putting the eligible candidates into either of the following trios: group A (vilazodone: 20-40 mg), group B (escitalopram: 10-20 mg), or group C (vortioxetine: 5-20 mg). The allocation ratio was 1:1:1. With blocks of sizes 12 and 24, we adopted permuted block randomization. As per the gender and duration of MDD, the randomization was stratified. We planned to evaluate the comparative efficacy and safety of three antidepressant medications - vilazodone, escitalopram, and vortioxetine - as monotherapy for the treatment of MDD over 16 weeks. The primary objective was to ascertain the change in HDRS scores from baseline through week 16 for each treatment group. Secondary objectives focused on assessing the change in MADRS score from baseline through week 16 and the incidence and severity of adverse events associated with each antidepressant medication. Specifically, the ITT and PP populations constituted the subjects for the safety and efficacy evaluations.

The participants received their prescribed medications as monotherapy for the entire trial duration. All of them were dosed with oral tablets once a day for 16 weeks. Other antidepressants or crossovers of study medications were prohibited. The psychiatrist customized the prescribed treatment dose in light of the individual's response to the medication. The baseline visit included a thorough physical and psychological evaluation of each participant.

Tools and assessments

Four, eight, 12, and 16 weeks after initiating the therapy, the participants were scheduled for follow-up appointments. The assessment of all efficacy and safety endpoints was part of each visit. We adopted HDRS and MADRS for the efficacy assessments [19,20]. Adverse event frequency and severity were determined as well. The severity of those events was assessed with Hartwig's severity scale. We additionally analyzed the causality of each documented event. Individuals who endured serious side effects or treatment failure were placed on rescue therapy, which entails conventional antidepressants. Treatment failure was outlined as increases of three or five points in the HDRS or MADRS scores from the previous visit.

Sample size calculation

We considered a mean change of 10.0 in HDRS compared to the baseline plus a standard deviation (SD) of 2.0 when computing the sample size. We needed 87 patients with 80% power and a 0.05 two-sided alpha error. We ultimately decided on 96 cases as the sample size to accommodate a 10% attrition rate. We ran an interim analysis after completing the 12th-week visits for the first 48 participants.

Statistical analysis

To verify that the data collected were normally distributed, we implemented the Shapiro-Wilk test. For categorical variables, the summary statistics were frequency and proportion. The median and interquartile range (IQR), or mean and SD, were used to convey the continuous data. We juxtaposed the sociodemographic traits using Pearson's chi-square test. The Kruskal-Wallis test was calibrated to gauge the median HDRS and MADRS scores. The Bonferroni test was adopted in post-hoc analysis. For data analysis, we employed R (4.3.3) [21]. The statistical tests were two-tailed. P-values below 0.05 were elucidated as statistically significant.

## Results

**Figure 1:**
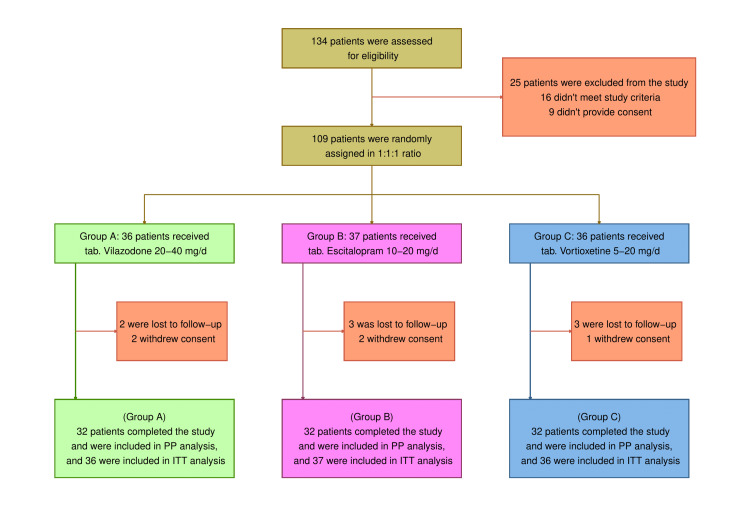
CONSORT diagram CONSORT: consolidated standards of reporting trials; ITT: intent-to-treat analysis; PP: per-protocol analysis

A total of 134 patients underwent the eligibility screening process (Figure 1). The study excluded 25 subjects. Nine refused participation, while sixteen fell short of the criteria. In total, 109 patients were allocated randomly into either of the three study groups. Five withdrew their permission, and eight failed to follow up. To evaluate the efficacy endpoints, 96 participants who belonged to the PP population were analyzed. The ITT population consisted of 109 patients who underwent assessment for safety. All three groups' participants had comparable baseline demographic characteristics (Table 1).

**Table 1:** Baseline traits of the PP population (n = 96) n (%) was used to display the category values. The median (IQR) or the mean ± SD were chosen to depict the continuous variables. MADRS: Montgomery Åsberg Depression Rating Scale; HDRS: Hamilton Depression Rating Scale; BMI: body mass index; T/T naïve: a newly diagnosed or treatment-naïve patient; PP: per-protocol

	Total (n = 96)	Group A: vilazodone (n = 32)	Group B: escitalopram (n = 32)	Group C: vortioxetine (n = 32)	p-value
Age (years)	46.3 ± 6.2	47.1 ± 6.4	46.0 ± 5.5	45.7 ± 6.1	0.143
Age group					
≤50 years	64 (66.7%)	23 (71.9%)	20 (62.5%)	21 (65.6%)	0.580
>50 years	32 (33.3%)	9 (28.1%)	12 (37.5%)	11 (34.4%)	
Gender					
Female	48 (50.0%)	16 (50.0%)	16 (50.0%)	16 (50.0%)	1
Male	48 (50.0%)	16 (50.0%)	16 (50.0%)	16 (50.0%)	
Marital status					
Married	72 (75.0%)	25 (78.1%)	23 (71.9%)	24 (75.0%)	0.477
Unmarried	24 (25.0%)	7 (21.9%)	9 (28.1%)	8 (25.0%)	
Education level					
Literate	80 (83.3%)	27 (84.4%)	26 (81.2%)	27 (84.4%)	0.189
Illiterate	16 (16.7%)	5 (15.5%)	6 (18.8%)	5 (15.5%)	
Duration of disease					
T/t naïve	48 (50.0%)	16 (50.0%)	16 (50.0%)	16 (50.0%)	1
<6 months	48 (50.0%)	16 (50.0%)	16 (50.0%)	16 (50.0%)	
BMI (kg/m^2^)	27.3 ± 4.8	26.4 ± 4.1	27.7 ± 5.2	27.8 ± 4.5	0.028
HDRS	30.05 ± 1.52	30.06 ± 1.50	29.94 ± 1.34	30.16 ± 1.74	0.964
MADRS	35.73 ± 1.47	35.81 ± 1.60	35.81 ± 1.23	36.03 ± 1.67	0.741

**Figure 2:**
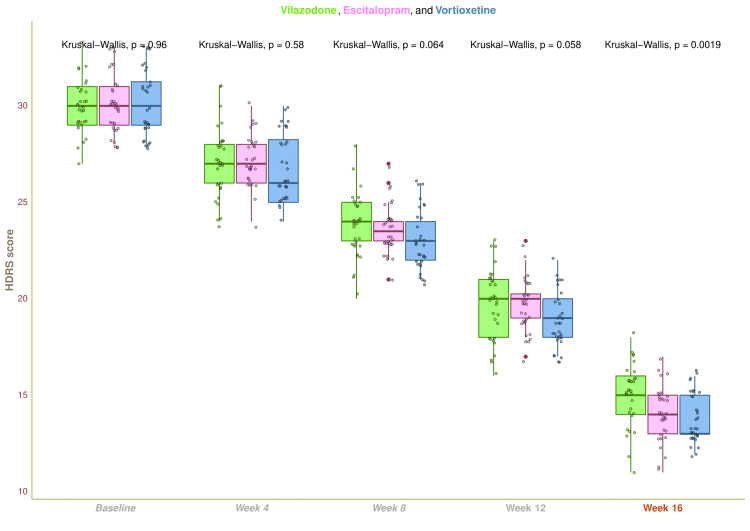
HDRS scores of participants at various time points of assessments The HDRS scores for the three groups' participants are depicted through the box-whisker and jitter plots. During every visit, the Kruskal-Wallis test was applied to assess the groups collectively. HDRS: Hamilton Depression Rating Scale

**Figure 3:**
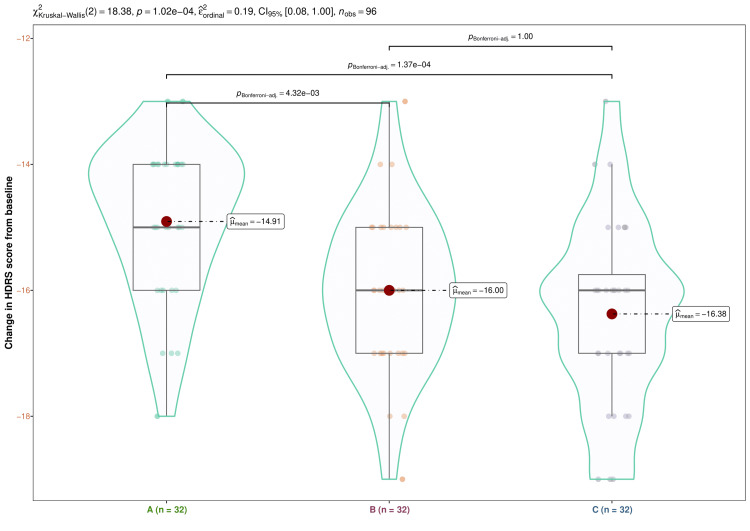
Post-hoc analysis of the differences in HDRS scores from baseline Changes in HDRS values from the baseline for the three groups' subjects are displayed via the box-whisker, jitter, and violin plots. The mean changes are highlighted through the red dots. The Bonferroni test was used after the Kruskal-Wallis test for the intergroup comparison. HDRS: Hamilton Depression Rating Scale

The three study groups' median baseline HDRS scores were 30.0 (29.0-31.0), 30.0 (29.0-31.0), and 30.0 (29.0-31.2), respectively (p = 0.964). Following four weeks of treatment, the corresponding scores were 27.0 (26.0-28.0), 27.0 (26.0-28.0), and 26.0 (25.0-28.2) (p = 0.583). The medians became 24.0 (23.0-25.0), 23.5 (23.0-24.0), and 23.0 (22.0-24.0), respectively, after an eight-week interval (p = 0.064). After 12 weeks, the figures were as follows: 20.0 (18.0-21.0), 20.0 (19.0-20.2), and 19.0 (18.0-20.0) (p = 0.058). The HDRS scores at the 16-week visit were 15.0 (14.0-16.0), 14.0 (13.0-15.0), and 13.0 (13.0-15.0), respectively (p = 0.002). In each of our study groups, we detected a statistically significant reduction in HDRS scores (p < 0.001) (Figure 2). These results imply that following a 16-week intervention, the study population had a decrease in the quantity, intensity, and frequency of symptoms of depression. Statistically significant differences (p < 0.001) were seen in intergroup comparisons. We conducted the post-hoc analysis using the Bonferroni correction. It disclosed that there was a statistically significant difference (p < 0.001) between the groups receiving escitalopram and vortioxetine and the group receiving vilazodone (Figure 3).

**Figure 4:**
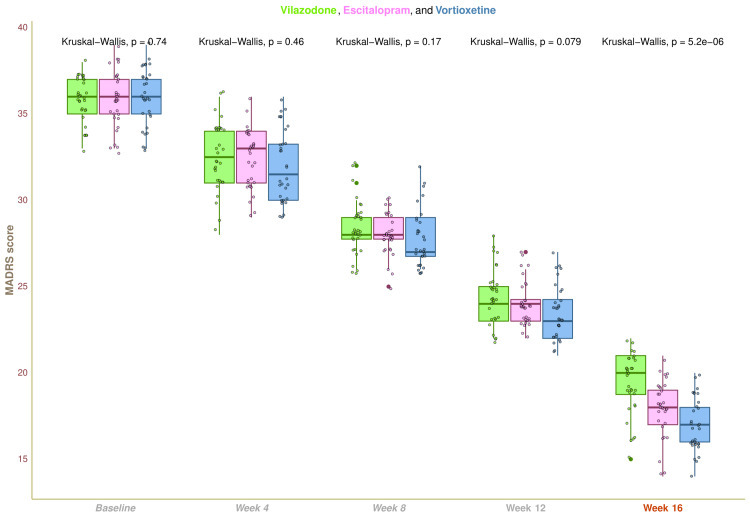
MADRS scores of participants at various time points of assessments The MADRS scores for the three groups' participants are depicted through the box-whisker and jitter plots. During every visit, the Kruskal-Wallis test was applied to assess the groups collectively. MADRS, Montgomery Åsberg Depression Rating Scale

**Figure 5:**
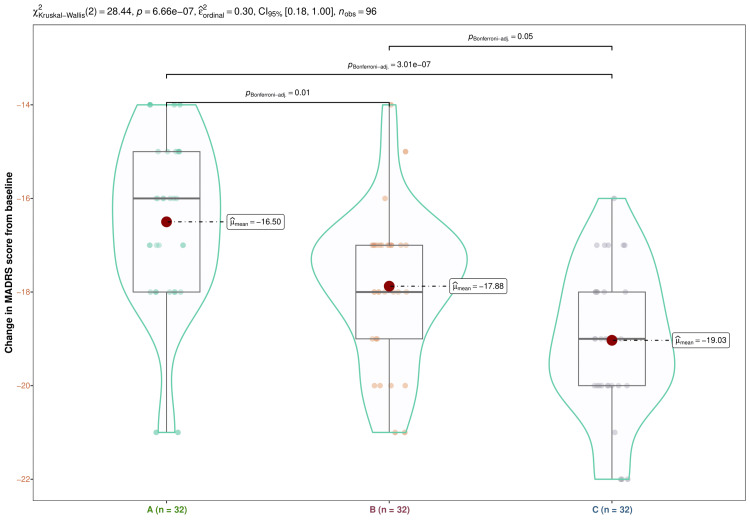
Post-hoc analysis of the differences in MADRS scores from baseline Changes in MADRS values from the baseline for the three groups' subjects are displayed via the box-whisker, jitter, and violin plots. The mean changes are highlighted through the red dots. The Bonferroni test was used after the Kruskal-Wallis test for the intergroup comparison. MADRS, Montgomery Åsberg Depression Rating Scale

The median MADRS score at the baseline visit was 36.0 (35.0-37.0) (p = 0.741) for the three research groups and the entire study population. The three groups' respective median scores after four weeks of therapy were 32.5 (31.0-34.0), 32.5 (31.0-34.0), and 31.5 (30.0-33.2) (p = 0.462). The MADRS scores at eight weeks were 28.0 (27.7-29.0), 28.0 (27.7-29.0), and 27.0 (26.7-29.0), respectively (p = 0.174). After twelve weeks, the scores were 24.0 (23.0-25.0), 24.0 (23.0-24.2), and 23.0 (22.0-24.2), in that order (p = 0.079). Following a 16-week visit, the MADRS scores were 20.0 (18.7-21.0), 18.0 (17.0-19.0), and 17.0 (16.0-18.0), in that order (p < 0.001). During 16 weeks of treatment, the MADRS scores for all three study groups significantly declined (p < 0.001) (Figure 4). An inter-group analysis found a statistically significant difference (p < 0.001) between the three study arms using the Kruskal-Wallis test. So, utilizing the Bonferroni correction, we carried out the post-hoc analysis. It indicated that the vortioxetine and vilazodone groups showed the most significant difference (p < 0.001) (Figure 5).

**Figure 6:**
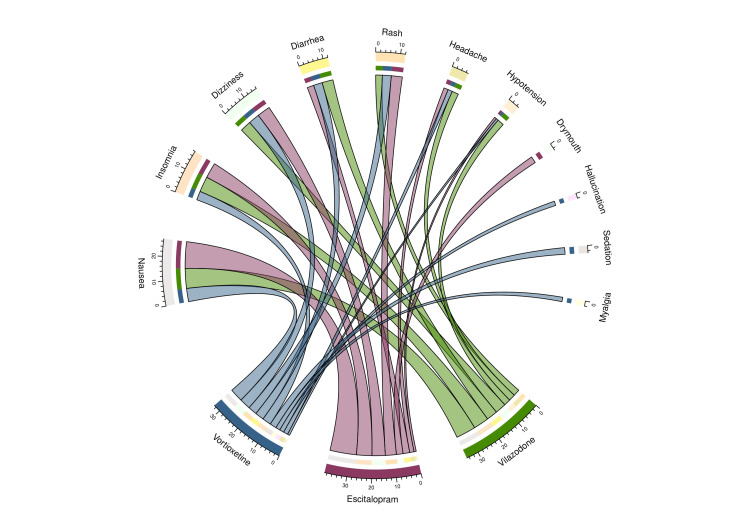
Adverse events noted in the study participants The lower portion of the plot illustrates the three drug groups, and the upper portion describes the different types of adverse events noted by the participants. The bands between the lower and upper sections indicate all the events observed in each group. The number of adverse events associated with the three study groups correlates precisely with the bandwidths.

An intent-to-treat (ITT) analysis was utilized to evaluate the adverse events encountered by every participant, as illustrated in Figure 6. There were 96 adverse events reported. The escitalopram group's participants encountered the highest number of incidents (39), followed by the groups on vilazodone (33) and vortioxetine (24). Based on NCI-CTCAE version 5.0, none of the incidents appeared significant. In line with Hartwig's severity scale, most events (76) were moderate. Four occurrences were considered severe, while 16 were classified as moderate. Adverse events led five participants to quit the trial. These included diarrhea (two in the vilazodone group and one in the escitalopram group), sleep difficulties (one in the escitalopram group), and skin rash (one in the vortioxetine group) as reasons for stopping the medication. The drugs, i.e., vilazodone, escitalopram, and vortioxetine, caused seven, eight, and 10 different types of adverse events, respectively.

Nausea and vomiting (24), insomnia (17), feeling lightheaded (15), gastroenteritis (11), rashes (10), headache (7), and hypotension (5) were the most frequently encountered adverse reactions in the study population. Two individuals on vortioxetine reported experiencing occasional visual hallucinations, whereas three participants on escitalopram complained of dry mouth. Adverse effects such as myalgia and extended sleep duration were noticed solely in the vortioxetine group (Table 2).

**Table 2:** Adverse events in the ITT population (n = 109) Using Hartwig's severity scale and NCI-CTCAE version 5.0, respectively, the seriousness and severity of all the adverse events were assessed. We used the chi-square (χ2) test to compute the p-values. NCI-CTCAE: National Cancer Institute Common Terminology Criteria for Adverse Events; NA: not applicable; ITT: intent-to-treat

	Group A: vilazodone (n = 36)	Group B: escitalopram (n = 37)	Group C: vortioxetine (n = 36)	p-value
Total adverse events	33	39	24	0.013
Serious (> grade 3)	0	0	0	NA
Severity of events				0.034
Mild	26	31	19	
Moderate	6	6	4	
Severe	1	2	1	
Causality of events				0.003
Probable	5	7	2	
Possible	24	27	26	
Unlikely	4	3	8	
Events led to discontinuation	2	2	1	0.671
Individual events				
Nausea and vomiting	8	12	4	< 0.001
Sleep disturbances	6	8	3	0.042
Dizziness	5	6	4	0.094
Diarrhea	5	3	3	0.060
Skin rash	3	4	3	0.068
Headache	3	2	2	0.119
Hypotension	3	1	1	0.053
Dry mouth	0	3	0	NA
Hallucination	0	0	2	NA
Sedation	0	0	1	NA
Myalgia	0	0	1	NA

## Discussion

This study found that, in contrast to vilazodone, a 5-HT1A receptor partial agonist, and escitalopram, an SSRI, vortioxetine, a serotonin receptor modulator, significantly decreased HDRS and MADRS scores at 16 weeks. Most of the adverse events were mild and non-serious. Patient tolerance and safety profiles were better in the vortioxetine group. These results concur with this study’s interim analysis and two recent meta-analyses [3,7,9,14,17,18].

The two experimental groups received vilazodone (20-40 mg daily) and vortioxetine (5-20 mg daily). A daily dose of 10-20 mg of escitalopram was administered to those who were recruited for the control group. Since vilazodone is a partial agonist targeting the 5-HT1A receptor, it brings a further edge over escitalopram, which has a single mode of action as an SSRI. On the other hand, vortioxetine directly affects serotonin receptors and impedes serotonin transport. Compared to the other two trial groups, the vortioxetine group experienced statistically significant reductions in HDRS and MADRS scores owing to its mechanisms. These results suggest that vortioxetine monotherapy could be an effective option for addressing MDD.

All participants received free drugs during the trial, regardless of their assigned groups. The dramatic decline in scores and the progressive amelioration of depressive symptoms over time imply that this may have enabled a low attrition rate. It could have boosted the antidepressant effects of the medications. HDRS and MADRS scores, as well as the diversity of adverse events, were statistically significantly better in the vortioxetine group. Vortioxetine could make a difference in MDD management in the upcoming years [3,18]. Optimizing antidepressant effects requires a lower attrition rate, a multi-tool evaluation of depression-related symptoms, and a lower incidence of adverse events, according to a recent network meta-analysis [17]. Because every facet is covered in this study, the findings may make sense. The findings are consistent with the interim analysis of this study [3,7,9,14].

The trial's key strengths were using permuted block randomization and assessing MDD with two well-known instruments, namely HDRS and MADRS [19,20]. Added advantages were routine, periodic checkups, and assessments of the ITT and PP populations. There is room for improvement in a few other areas of our study. At the outset, the open-label trial design may influence the dropout rate and inherent reporting biases regarding adverse events. Second, the study's antidepressants were provided free of charge. The study's findings only apply if these medications are affordable and readily available. Third, depression has several facets and underlying causes. Determining the efficacy of long-term antidepressant therapy in a real-world situation is challenging.

## Conclusions

When juxtaposed with vilazodone and escitalopram, vortioxetine provided significant reductions in the HDRS and MADRS scores. It also had a better safety profile. The observed differences in efficacy and safety might influence clinical decision-making and treatment guidelines for MDD. In the future, blinded trials might improve the robustness of the data, providing a more nuanced perspective on the implications of the study findings. Further studies exploring the antidepressant properties of these drugs should be conducted with a larger sample size.

## References

[1] Proudman D, Greenberg P, Nellesen D (2021). The growing burden of major depressive disorders (MDD): implications for researchers and policy makers. Pharmacoeconomics.

[2] Flux MC, Lowry CA (2020). Finding intestinal fortitude: integrating the microbiome into a holistic view of depression mechanisms, treatment, and resilience. Neurobiol Dis.

[3] Santi NS, Biswal SB, Naik BN, Sahoo JP, Rath B (2023). An interim analysis of a randomized, open-label study of vilazodone, escitalopram, or vortioxetine for major depressive disorder. Cureus.

[4] Bengoechea-Fortes SP, Ramírez-Expósito MJ, Martínez-Martos JM (2023). Suicide, neuroinflammation and other physiological alterations. Eur Arch Psychiatry Clin Neurosci.

[5] Liu Q, He H, Yang J, Feng X, Zhao F, Lyu J (2020). Changes in the global burden of depression from 1990 to 2017: findings from the Global Burden of Disease study. J Psychiatr Res.

[6] Sagar R, Dandona R, Gururaj G (2020). The burden of mental disorders across the states of India: the Global Burden of Disease Study 1990-2017. Lancet Psychiatry.

[7] Santi NS, Biswal SB, Naik BN, Sahoo JP, Rath B (2023). Quality of life and medication adherence in patients with major depressive disorder: an interim analysis of a randomized study. Cureus.

[8] Qiu W, Cai X, Zheng C, Qiu S, Ke H, Huang Y (2021). Update on the relationship between depression and neuroendocrine metabolism. Front Neurosci.

[9] Santi NS, Biswal SB, Naik BN, Sahoo JP, Rath B (2023). Metabolic effects of antidepressants: results of a randomized study’s interim analysis. Cureus.

[10] Ormel J, Spinhoven P, de Vries YA, Cramer AO, Siegle GJ, Bockting CL, Hollon SD (2020). The antidepressant standoff: why it continues and how to resolve it. Psychol Med.

[11] Pastoor D, Gobburu J (2014). Clinical pharmacology review of escitalopram for the treatment of depression. Expert Opin Drug Metab Toxicol.

[12] Wang SM, Han C, Lee SJ, Patkar AA, Masand PS, Pae CU (2016). Vilazodone for the treatment of depression: an update. Chonnam Med J.

[13] Connolly KR, Thase ME (2016). Vortioxetine: a new treatment for major depressive disorder. Expert Opin Pharmacother.

[14] Santi NS, Biswal SB, Naik BN, Sahoo JP, Rath B (2023). Comparison of Hamilton Depression Rating Scale and Montgomery-Åsberg Depression Rating Scale: baked straight from a randomized study. Cureus.

[15] Lee SH, Jeon SW, Shin C (2022). Acute efficacy and safety of escitalopram versus desvenlafaxine and vortioxetine in the treatment of depression with cognitive complaint: a rater-blinded randomized comparative study. Psychiatry Investig.

[16] Bathla M, Anjum S (2020). A 12-week prospective randomized controlled comparative trial of vilazodone and sertraline in Indian patients with depression. Indian J Pharmacol.

[17] Kishi T, Ikuta T, Sakuma K, Okuya M, Hatano M, Matsuda Y, Iwata N (2023). Antidepressants for the treatment of adults with major depressive disorder in the maintenance phase: a systematic review and network meta-analysis. Mol Psychiatry.

[18] Milosavljević F, Molden PE, Ingelman-Sundberg PM, Jukić AP (2024). Current level of evidence for improvement of antidepressant efficacy and tolerability by pharmacogenomic-guided treatment: a systematic review and meta-analysis of randomized controlled clinical trials. Eur Neuropsychopharmacol.

[19] Hamilton M (1960). A rating scale for depression. J Neurol Neurosurg Psychiatry.

[20] Montgomery SA, Asberg M (1979). A new depression scale designed to be sensitive to change. Br J Psychiatry.

[21] (2024). R: A language and environment for statistical computing, Vienna, Austria. https://www.r-project.org/.

